# A Brief History of Charcot-Leyden Crystal Protein/Galectin-10 Research

**DOI:** 10.3390/molecules23112931

**Published:** 2018-11-09

**Authors:** Jiyong Su

**Affiliations:** Jilin Province Key Laboratory on Chemistry and Biology of Natural Drugs in Changbai Mountain, School of Life Sciences, Northeast Normal University, Changchun 130024, China; sujy100@nenu.edu.cn; Tel.: +86-431-8509-8908

**Keywords:** Charcot-Leyden crystal protein, Galectin-10, history, cellular distribution, diseases marker, crystal structure, ligand binding specificity

## Abstract

Eosinophils are present in tissues, such as the respiratory tract, spleen, lymph nodes and blood vessels. The significant presence of eosinophils in these tissues are associated with various diseases, including asthma, allergies, acute myeloid leukemia, etc. Charcot-Leyden crystal protein/galectin-10 is overexpressed in eosinophils and has also been identified in basophils and macrophages. In human body, this protein could spontaneously form Charcot-Leyden crystal in lymphocytes or in the lysates of lymphocytes. At present, the role of Charcot-Leyden crystal protein/galectin-10 in lymphocytes is not fully understood. This review summarizes research progress on Charcot-Leyden crystal protein/galectin-10, with emphasis on its history, cellular distributions, relations to diseases, structures and ligand binding specificity.

## 1. Charcot-Leyden Crystals

Charcot-Leyden crystals (CLCs), having both hexagonal and bipyramidal forms, were first reported in 1853 by Jean-Martin Charcot who found tiny crystals in the cardiac blood and spleen of a patient who died from leukemia [1]. In 1872, Ernst Viktor von Leyden also described colorless crystals found in the sputum of asthma patients [2]. Following the discovery of CLCs, many conflicting reports appeared as to about the chemical nature and significance of these crystals [3,4,5,6]. At that time, it was unknown that CLC was crystallized protein. CLCs were even incorrectly considered to be the same as prostatic secretion crystals or ion crystals [7,8]. In 1895, Cohn correctly concluded that CLCs found in leukemic blood, in asthmatic sputum, in nasal polyps and in bone marrow were the same and differed from those found in prostatic secretion [9]. In 1914, the relationship of CLCs and eosinophils was reported by Schwarz [6], a proposal that facilitated studies of CLCs.

Early work demonstrated that CLCs could only be found in humans and primates [5,10]. CLCs could not form in guinea pig eosinophils [11], and investigation of horse and rabbit blood, upon various stimuli, also yielded no crystals [5]. However, one type of four-sided crystal was observed in eosinophils from mice with pneumonia [12] and in the macrophages of a mouse asthma model [13]. However, these crystals are composed of a chitinase-like protein, Ym1, but not CLC protein [14].

The primary structures of human and primate CLC protein are more similar to each other than to other species [15]. Even though it appears that CLCs only form in humans and monkeys, it is curious that they are not found in other species, especially since no reliable techniques like mass spectroscopy have been used to determine the actual composition of protein crystals found in these species. 

## 2. Ambiguous History of CLC Protein/Galecin-10

Before the CLC protein gene was sequenced, it was inappropriately considered to be a lysophospholipase [16,17]. While a 74 kDa enzyme was found to be the actual lysophospholipase [18], a pull-down assay showed that CLC protein could interact with this lysophospholipase [19]. This indicates that the initial enzyme assay for CLC protein was actually contaminated by lysophospholipase.

**Figure 1:**
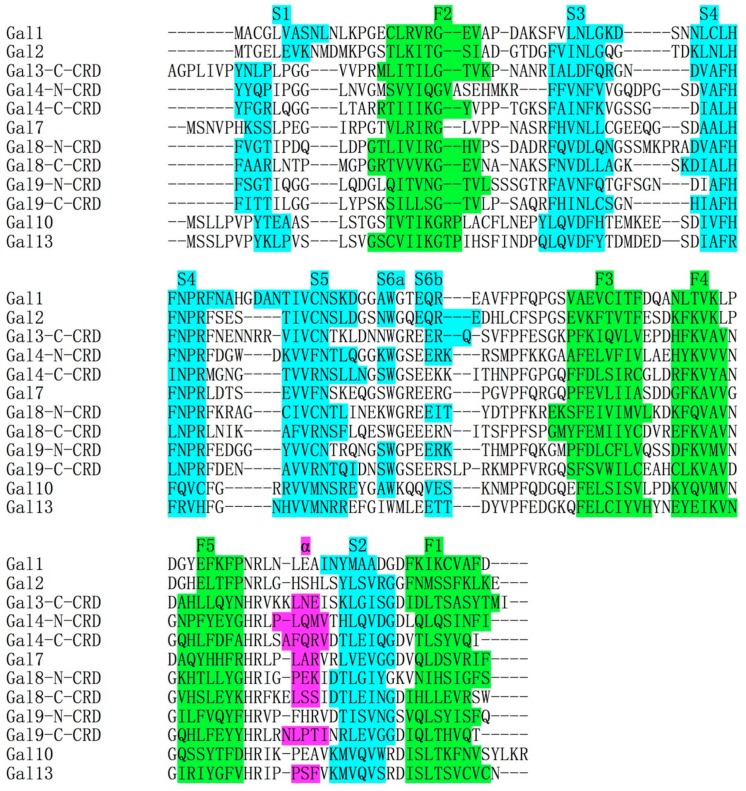
Amino acid alignment of carbohydrate binding domain (CRD) of several human galectins. Beta strands in S-face and F-face were colored by cyan and green, respectively. A small alpha helix (colored by purple) occasionally forms in several galectin CRDs. The primary structure of Gal-10 is homologous to the CRDs of other galectins. The alignment was generated by CLUSTAL 2.1. The primary structure of Gal-12 was not used in this alignment because the secondary structure of Gal-12 was not determined.

The CLC protein gene was mapped to human chromosome 19 [20] and later more accurately mapped to chromosome 19q13.2 by the Human Genome Project (http://www.genecards.org). The intron–exon architecture of the CLC protein gene is similar to that of galectins, with the carbohydrate binding domain (CRD) encoded by a single exon [21]. The primary structure alignment of the CLC protein amino acid sequence with other galectins showed that the CLC protein was homologous to the carbohydrate binding domain (CRD) (Figure 1) [22]. Moreover, the CLC protein crystal structure showed that it has a highly similar fold to galectin CRD [23]. Because of this, the CLC protein was re-named galectin-10 (Gal-10) [24]. In the asymmetric unit of the Gal-10 crystal, there is only one Gal-10 monomer, a pseudomorph that did not accurately display the global form of Gal10. Actually, Gal-10 is a homodimeric protein in solution [25]. 

Until now, the natural ligands of Gal-10, including proteins and carbohydrates, have not been fully identified. However, there are several contradictory results about the ligand binding specificity of Gal-10. A solid phase assay showed that Gal-10 could bind weakly to lactose and *n*-acetyl-*d*-glucosamine conjugated to agarose [23,26]. However, three contradictory results showed that the weak carbohydrate binding activity may be attributed to wild type Gal-10 binding to the cross-linked agarose (or Sepharose) matrix and not to lactose [19,25,27], and the crystal structure of Gal-10 showed that it could bind mannose [23]. Recently, we generated a variant of Gal-10 (W127A) that exists as a monomer and can weakly bind to lactose-modified Sepharose [28].

CLCs isolated from human eosinophils contains 1.2% carbohydrate content [29]. The mass spectroscopy of Gal-10 obtained from nasal fluid showed that Gal-10 was not glycosylated but could be acetylated at Ser2 [30]. High resolution crystal structure of Gal-10, which was purified from a human myeloid leukemic cell line, also showed absence of glycosylation [23]. Overall, Gal-10 is not glycosylated and can weakly bind to carbohydrate under several conditions, like in its monomeric state. 

## 3. Crystallization Studies of Gal-10

After the discovery of CLCs, optimization of Gal-10 crystallization in various detergents and surfactants was performed [31,32,33]. These studies showed how fast Gal-10 could crystallize. Using the surfactant Aerosol MA as the cell lysates of eosinophilia, large numbers of CLCs could be formed within seconds [32]. Synthetic anionic, cationic and non-ionic detergents could also produce rapid formation of CLCs from eosinophils [33]. Crystallization studies facilitated the subsequent 3-D structure determination of Gal-10 [19,23,34]. One study showed that addition of substances including serum plasma and breakdown products from red blood cells could prevent formation of CLCs [31]. These results implied that Gal-10 should bind to intracellular partners and that the crowded intracellular environment might restrict intermolecular interactions with Gal-10 to inhibit crystallization.

## 4. Structural Comparisons between Gal-10 and Other Prototype Galectins

Since the discovery of Gal-1, fourteen genes of galectins have been found in the human genome [35,36,37,38]. Although human galectins lack a signal peptide sequence, they can be transported from the cytoplasm by an unknown pathway. A recent report showed that Gal-3 was secreted from the cytoplasm via microsome extrusion [39]. Upon apoptosis or necrosis, galectins are also released and regulate the functions of nearby cells [38]. In the extracellular matrix, galectins bind to β-galactoside units in polysaccharides, glycosylated extracellular proteins and glycosylated lipids. Galectins are also involved in many physiological functions, such as inflammation, immune responses, cell migration, autophagy and signaling. They are also associated with many diseases, such as fibrosis, cancer and heart disease [38]. 

Based on structural differences, galectins are grouped into three types: tandem-repeat, chimeric, and prototype [37,40,41,42,43]. Each of these types have distinctive global structures. Tandem-repeat-type galectins, including galectin-4, -8, -9 and -12, have two CRDs connected by a short linker peptide [43]. So far, the structure of any full length tandem-repeat-type galectin has yet to be solved primarily due to its inherent flexibility of the structure. Variants of Gal-8 and -9 with shortened liker peptides, however, could be crystallized, and their structures were solved [44,45].

Gal-3, the only chimeric type galectin, has a long proline-rich repeat, collagen-like N-terminal tail [46]. Even though many crystal structures of the Gal-3 CRD have been reported [47,48,49,50], its N-terminal tail is highly flexible and interacts transiently with the F-face of the CRD [51], a dynamic situation that precludes crystallization of the full-length lectin. Recently, an interesting crystal structure of Gal-3 was published showing that a short peptide from Gal-3 N-terminal tail can extend the CRD F-face β-sheet [52]. 

Although the structures of some prototype galectins (i.e. galectin-14, -16 and -17) have yet to be reported, most prototype galectin structures are known [34,53,54,55,56]. Crystallographic and biochemical analyses show that Gal-1, -2, -7 and -10 form dimers via non-covalent interactions [34,53,54,55].

The global conformations of Gal-1 and Gal-2 are quite similar with a β-sandwich structure with the same number of beta strands [53,54]. The carbohydrate bindings sites of these galectins are located at the opposite sites in each CRD. The crystal structure of Gal-1 shows that each CRD binds one molecule of lactose, indicating that it has a functional role. Because the crystallization time is relatively long, binding of lactose to the two CRDs may not occur independently. In fact, NMR results demonstrated that binding of one lactose molecule to a Gal-1 CRD can negatively regulate the binding of the other [57]. In the crystal structure of Gal-2, one lactose molecule binds to one CRD, and yet the other site was found to be occupied by an aspartate residue (Asp29) [54]. This is similar to the way in which Gal-1 binds lactose in solution. However, because of this, Gal-1 ligand binding specificity is different from that of Gal-2, implying that these two galectins have different functions in vivo. 

Gal-1 purified from eukaryotic cells has a higher molecular weight than Gal-1 purified from *E. coli* [58]. This implies that Gal-1 is chemical modified in vivo. Moreover, at low concentration (7 μM), Gal-1 dimers can disassociate, with its ligand binding specificity being different from Gal-1 dimers [59]. In contrast, it has been reported that Gal-1, even at 2 μM, maintains the dimeric state [60,61] as shown by NMR structural studies of Gal-1 in solution [57]. A Gal-1 variant with double mutations (C2S/V5A) has been shown to exist as a monomer [62], with its sugar binding ability being lower than that of the wild type lectin [59]. Gal-1 has six cysteine residues that can be oxidized [53]. The oxidized form of Gal-1 has lower sugar binding specificity compared to the fully reduced state [59]. The main function of oxidized Gal-1 has been suggested to regulate signal transduction in cells [63,64]. Furthermore, reduced Gal-1 can bind to galactose residues of glycans within the extracellular matrix and thus could trigger signal transduction pathways from outside to inside of the cell [59].

Gal-7 forms dimers via its F face, with several residues forming hydrogen and ionic bonds, as well as van der Waals interactions that promote its dimeric structure. Two Gal-7 monomers can bind to two molecules of galactose [55].

**Figure 2:**
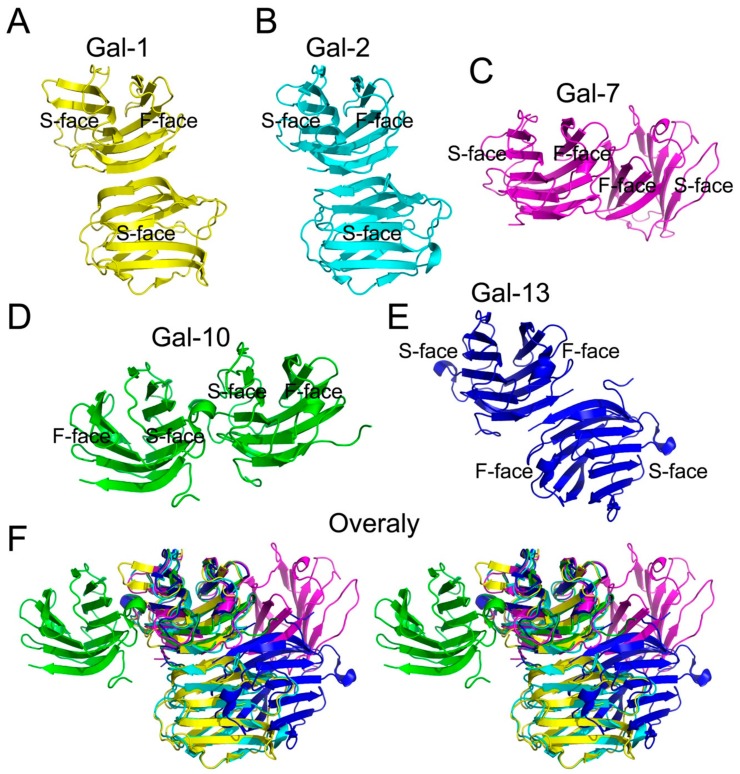
Human prototype galectins with different global forms. (**A**) Gal-1 dimeric structure; (**B**) Gal-2 dimeric structure; (**C**) Gal-7 dimeric structure; (**D**) Gal-10 dimeric structure; (**E**) Gal-13 dimeric structure; (**F**) stereoscopic overlay of Gal-1, -2, -7, -10 and -13. Each human prototype galectin has two CRDs and each CRD has one S-face and one F-Face. Besides Gal-1 and Gal-2, the dimeric packing patterns of the other three prototype galectins are different from each other.

Gal-10 is highly expressed in eosinophils [29]. The first structure of Gal-10, solved by using Gal-10 purified from a human myeloid leukemic cell line [23], showed that there is only one Gal-10 monomer in each asymmetric unit. Recently, we and Kamitori used PISA to predict interactions between two Gal-10 CRDs in two separate asymmetric units close in proximity and proposed that Gal-10 may dimerize [25,40]. This model of Gal-10 has a S-face to S-face structure and is significantly different from other prototype galectins (Figure 2). Gel filtration validated the formation of Gal-10 dimers in solution [25]. In addition, a key residue (Trp127) helps mediate Gal-10 dimerization [28]. In fact, mutation of Trp127 to alanine abolishes Gal-10 dimer formation. 

Gal-13 is an unusual prototype galectin in that it forms dimers via disulfide bonds formed between two cysteine residues (Cys136 and Cys138) at the C-terminus of its CRD [56]. A variant with two mutations (C136S/C138S) exists as a monomer in solution [56]. Hemagglutination assays have shown that DTT could inhibit this variant inducing erythrocyte agglutination, indicating that the Gal-13 dimer structure is crucial to its function. 

The structural arrangements of Gal-7, -10 and -13 are different from Gal-1 and Gal-2 (Figure 2). Although there is consensus that all galectins evolved from a single ancestor protein, the global structure of these lectins has changed during evolution, along with their respective functions. Taken together, this suggests that all galectins have different global forms and functions, although there is no clear answer as to why and how these prototype galectins have obtained different structures.

## 5. Ligand Binding Specificity of Gal-10

Just after completion of the Gal-10 cDNA sequence [22], Dyer et al., found that the primary structure of Gal-10 is highly similar to Gal-1 and Gal-3 [26]. Thus, it has been assumed that Gal-10 is a member of galectin family [21]. The solid assay can be used to test the capacity of Gal-10 binding to lactose-modified agarose. The results showed that Gal-10 can only weakly bind to these beads [23,26]. However, three other reports contradict this conclusion [19,25,27]. In addition, the crystal structure showed that Gal-10 can co-crystallize with mannose, but not lactose [23]. However, the variant (W127A), which exists as a monomer, can bind to lactose-modified agarose beads [28]. By investigating the homodimeric structure of Gal-10, we found that a glutamate residue (Glu33) from the S-face of one monomer directly occupies the carbohydrate binding site of another monomer. This glutamate residue may inhibit lactose binding to Gal-10. A variant with a mutation from Glu33 to alanine was generated, and its crystal structure showed that the variant can be co-crystallized with lactose. Overall, the behavior of mutants E33A and W127A indicates that Gal-10 has a functional carbohydrate binding site, whereas the dimeric structure influences Gal-10 binding to β-galactosides. Gal-1 can disassociate into monomers [65]. Thus, Gal-10 dimers may also disassociate in the same way and can bind carbohydrates. However, this proposal requires further experimentation to be verified.

## 6. Staining Methods for CLC

If the sizes of cellular CLCs are large enough, the crystals can be directly observed by light microscopy [66,67], phase contrast microscopy [68]. In addition, because Gal-10 in CLCs contains aromatic residues, they can be directly visualized by using fluorescent microscopy [69]. However, because CLCs are normally small and colorless, it is difficult to directly observe them using light microscopy unless that are color stained. 

The first rabbit antisera to human Gal-10 was developed in 1980 [70]; this antibody was used for immunofluorescence staining [71] and TEM immunogold labeling [72]. Immunofluorescence staining of CLCs provided a clear observation of CLCs in human basophils [71]. In addition, May-Grünwald Giemsa staining and papanicolaou staining could give blue and orange color to CLCs, respectively [73,74,75]. CLCs could be clearly distinguished from other cell organelles by hematoxylin-basic fuchsin-picric acid staining [76,77]. Hematoxylin eosin purple staining was used to identify CLCs in granulocytic sarcoma and in bone marrow [78,79]. Wright Giemsa staining allows CLCs from macrophages to be distinguished from other cells [79,80,81]. Acid-fast-positive staining is a new method that can effectively stain CLCs and make them distinguishable from other cells [69].

## 7. Promoter Studies of Gal-10 Gene

Several compounds can regulate Gal-10 transcription in cells. Butyrate can upregulate its transcription in HL-60 pro-myelocytic leukemia cells [82]. Another study showed that a GC box upstream of the transcription start site (-44 to -50) actually controls induction of gene expression by butyrate [83]. In contrast, dimethyl sulfoxide inhibits Gal-10 transcription in blood cells [84], although the mechanism of this inhibition remains unknown.

The Gal-10 transcription initiation site was identified at the 43 bp upstream of the 5′ end of the cDNA sequence. The cDNA upstream sequence (-1 to -411) contains consensus binding sites for several transcription factors, including GATA-1, PU.1, Oct, and Sp.1 [82]. One further study showed that the -1 to -1504 region contains several other transcription factor binding sites, such as EoTF, NF-1, AP-2, AML1 that could up or down regulate the expression of Gal-10. GATA-1 and EoTF binding sites were found to be essential for full promoter activity [85]. Moreover, disruption of either the GC box (-44 to -50) or the Orc site (-255 to -261) decreased Gal-10 promoter activity. In addition, super-shift analysis demonstrated that transcription factors Sp1 and Oct1 could bind to the consensus GC box and Oct site, respectively [83]. A bioinformatics study showed that homozygotes for the minor allele of single nucleotide polymorphisms in the CLC promoter region may regulate Gal-10 expression and thus might cause allergic rhinitis [86]. 

## 8. Distribution of Gal-10 in Variable Lymphocytes

Initially, Gal-10 was believed to be distributed only in eosinophils [3,4,5,6,7,8,9,10,11]. However, radioimmunoassays showed that extracts from basophils contained high levels of Gal-10 [71]. Co-cultures of eosinophils and macrophages demonstrated that macrophages could also endocytosis Gal-10 to form CLCs in the phagosomes of macrophages [27]. In T-cells, Gal-10 is also expressed, and has been identified as a marker for anergy and suppressive function of CD25+ Treg cells [87]. In patients with atopic dermatitis, the expression level of Gal-10 in circulating CD3+ T cells and IL-22-production in CD4+ T cells are elevated [88]. Eosinophils can even connect with the outer membrane of T cells with synapses that are partially constituted by Gal-10 [89].

## 9. Transmission Electron Microscopy Studies of CLCs

In the 1980s, transmission electron microscopy (TEM) was used to identify the distribution of Gal-10/CLCs in lymphocytes [72]. The resolution of these images was sufficient to analysis the distribution of Gal-10/CLCs in cell organelles. The first TEM of Gal-10 showed that it was primarily localized to a minor (about 5%) sub-population of eosinophil primary granules. These membrane-bound cytoplasmic granules contain no crystalloid inclusion [72]. However, a contradictory report showed that primary granules also contain eosinophil peroxidase, a crystalloid inclusion forming protein [90]. TEM showed that Gal-10 was present in the nuclear matrix and extra-organellar cytoplasm of interleukin-5-stimulated mature eosinophils [91]. Extensive amounts of Gal-10 were also present in granule-poor and subplasma membranes of the cytoplasm in mature eosinophils [91,92]. Other assays demonstrated that release of eosinophil granule proteins, including Gal-10, can be selective, and is triggered by stimulation of one or several membrane receptors [93,94]. By using TEM, Arne Egesten et al., found that Gal-10 is present in a rare granular compartment from both immature and mature eosinophils [95]. 

TEM also showed that Gal-10 was present in the phagosomes of macrophages [77,91] and late endosomes [90]. Gal-10 could be stored in the granules of unstimulated human basophils [66]. Both Gal-10 positive and negative granules indicated the same characteristic particulate-like structure of the granular matrix, with both sharing the same membrane marker CD63 [90]. After *N*-Formylmethionine (fMet) peptide stimulated human basophils, Gal-10 was released through vesicles to the plasma membrane exhibiting piecemeal degranulation (PMD). In the beginning of PMD, Gal-10 still could be associated with the plasma membranes of degranulation channels [66]. During degranulation, basophils uptake released Gal-10 that was rapidly recycled into cells after fMet peptide challenge [66,96]. After the initial seconds of degranulation, CLC protein was secreted as crystals that are directly extruded from intra-granular locations in morphological phenotypes associated with anaphylactic degranulation (AND) [66]. Aside from granules, Gal-10 is also distributed in the nucleus (in the euchromatin) [90], cytoplasm, plasma membrane [97] and Golgi-associated smooth membrane-bound vesicles [98]. Overall, TEM is perhaps the best technique available to analyze the distribution of cellular Gal-10. A nice protocol for using TEM for basophils and mast cells has been summarized by Dvorak [99].

## 10. Cellular Distribution of CLCs and Gal-10

CLCs are present in nuclear chromatin, cellular membrane debris and numerous empty and partially empty granules from lysed eosinophils and intact phagosomes [77]. This indicates that CLC formation is only dependent on the concentration of Gal-10. When the local concentration of Gal-10 is higher than the concentration for crystallization, Gal-10 crystallizes. In addition, the distribution of CLCs in granules, nucleus and phagosomes indicates that there must be some key transport systems for transferring the protein into these organelles. 

Immunofluorescent microscopy of human peripheral eosinophils showed that there is a considerable amount of Gal-10 concentrated in the nucleus [66]. This study is consistent with Calafat et al.’s study that showed Gal-10 is mainly distributed in the nucleus of eosinophils [90]. Our recent study on Gal-10 indicates that the EGFP-tagged lectin is primarily distributed in the nucleus of HeLa cells [25], indicating that Gal-10 can be actively transported to the nucleus. This also implies that Gal-10 might play a role in regulating transcription or translation through interactions with unknown ligands. 

Many reports showed that Gal-10 could be distributed in the cytoplasm [91,92,97]. However, the function of Gal-10 in the cytoplasm remains unknown. In addition, Gal-10 is distributed in the regions of outer and inner cell membrane. Gal-10 binds to the outer membrane of T-cells and suppresses T-cell function [87]. The release of Gal-10 from eosinophils suggests that it is stimulated by triggers on the outer membrane [93,94]. Upon stimulation, Gal-10 is secreted via a degranulation pathway or directly extruded from the cytoplasm [66,96]. Moreover, eosinophil apoptosis and necrosis also can promote release of Gal-10. 

Overall, the distribution of Gal-10 in cells seems to be clear. However, the molecular mechanism as to how Gal-10 function is still essentially unknown, with many open questions as to what are the in vivo partners to which Gal-10 can bind, how Gal-10 is transported, whether Gal-10 has a role in signal transduction and so on. To answer these questions, it is crucial to identify the in vivo ligands of Gal-10 by using various techniques such as pull down, co-IP and yeast two-hybrid. 

## 11. Relations of CLC/Gal-10 to Variable Diseases

Several tissues and body fluids from disease states contain many infiltrated mature eosinophils and CLCs that could form where eosinophils cluster. Therefore, CLC appearance and disorder expression of Gal-10 may be potential markers for several diseases. Until now, CLCs have been found in lesions of patients with immune system diseases, tumors, skin diseases, asthma, infections and intestinal diseases.

### 11.1. CLCs in The Lymphoid Organs

Quantitative reverse transcription PCR has showed that Gal-10 mRNA is highly transcribed in bone marrow [35], implying that lymphocytes require Gal-10 in regulating the selection and maturity of lymphocytes. Disorders or pathological changes to bone marrow may cause massive proliferation of eosinophils and significant CLC formation. Recently, several independent case reports indicated that numerous CLCs are found in the bone marrow of patients with acute myeloid leukemia [100,101,102,103]. Moreover, in a patient with a FIP1L1-PDGFRA-positive myeloproliferative disorder, CLCs were seen in the areas with the highest concentrations of eosinophils in bone marrow [79]. Numerous CLCs were also seen in the bone marrow amid increased numbers of eosinophils and the presence of dysplastic granulopoiesis in male with chronic eosinophilic leukemia [81]. In addition, following maturation of eosinophils in bone marrow, CLCs would be transferred into the lymphatic system of patients with eosinophil disorders. Actually, Jean-Martin Charcot first found CLCs in the spleen and cardiac blood of a patient with leukemia [1]. Collectively, the presence of large numbers of CLCs in lymphoid organs indicates that there are some lesions in these tissues.

### 11.2. CLCs in Stroma of Solid Tumors

Eosinophils can infiltrate into solid tumors [104]. In stroma of mastocytomas, eosinophils are present, and CLCs are found there in macrophages [77]. Two cases of granulocytic sarcoma were found to contain numerous CLCs in foci of necrosis or within macrophages [78]. CLCs are also found in an eosinophilic pseudo-tumor of the liver [105]. The existence of CLCs in tumors indicates that eosinophils could affect tumor growth and/or function [106].

### 11.3. CLCs and Infections

Eosinophils can synthesize, store and secrete cytokines, chemokines, and growth factors that can kill helminths, fungus, virus and bacteria, but they can also harm host tissue [107]. Helminth parasite infection induces an immune response that is characterized by IgE antibody production, as well as tissue and blood eosinophilia proliferation [108]. Lesions of certain patients infected with helminths, including pork tapeworm, filarial worms or nonparasitic lesions, contain numerous eosinophils and CLCs [73]. Fine-needle aspiration cytology smears prepared from the hepatic space occupying lesions showed numerous CLCs along with eosinophilic infiltrates, indicating that the patient was infected by a helminth [109].

Fungus contributes to the development of variable diseases, such as sinusitis and otitis. Eosinophils may directly or indirectly react with the fungus [110]. In lesions of allergic fungal sinusitis, there are many alternating mucinous materials and inflammatory cell debris and abundant CLCs [111]. Numerous CLCs have been observed in eosinophilic otitis media from patients infected with fungi in the middle ear [112]. 

Eosinophils can interact with bacterial pathogens in variable ways. They can engulf bacterial organisms by phagocytosis [113,114]. From a patient infected by Aspergillus, cytological investigation of the eosinophilic pleural effusion showed that the pleural fluid contained numerous eosinophils and CLCs [105]. 

Eosinophils may also be important for the host response to viral infections, particularly for viral respiratory infections. Quantitative reverse transcription PCR showed that Gal-10 is downregulated in infants with respiratory syncytial virus bronchiolitis. However, the reason of CLC downregulation remains unknown [115].

### 11.4. CLCs and Celiac Disease

The normal gastrointestinal tract is populated by eosinophils that are present throughout the mucosa [116]. An eosinophilic infiltrate has also been described in the duodenal mucosa of patients with active celiac disease [117]. A duodenal biopsy specimen demonstrated a significantly higher expression of Gal-10 in a celiac disease patient [118]. Because of these studies, it has been suggested that Gal-10 is a novel marker for evaluating celiac disease tissue damage and eosinophils as a possible target for therapeutic approaches [119]. 

### 11.5. CLCs in Asthma and Allergy

Eosinophils play a key role in the symptoms of asthma and allergies. An infection can cause massive eosinophil development in the respiratory system of patients. A large presence of eosinophils may trigger asthma in patients [120]. Ernst Viktor von Leyden first described these colorless crystals in the sputum of asthma patients [2]. Blood leukocytes collected from a man with bronchial asthma and eosinophilic leukocytes contain approximately 75% eosinophils. The blood lysate can easily form CLCs [121]. In certain patients with both asthma and bronchopulmonary infection, the Gal-10 level was elevated [122], indicating that CLCs can be used as a marker of asthma relating to eosinophils. Eosinophil-rich inflammation has been associated with allergies [123]. In the tears of patients with vernal kerato-conjunctivitis, one type of allergy, CLCs have been found [124]. Gal-10 was also present in all samples from patients with seasonal allergic rhinitis during the allergy season, but not in any sample prior to allergy season [30].

### 11.6. CLCs Related to Other Diseases

CLCs have been found in the spun urine sediment of a patient with hypereosinophilic syndrome and acute renal failure [125]. CLCs were also found in cervical smears [126]. In the eosinophilic granuloma of bone, numerous CLCs exist [127]. CLCs were found in the synovium and the synovial fluid of a female [68]. TEM revealed the presence of CLCs within the periapical lesion of a patient [128]. The occurrence of CLC in skin lesions is very rare. The pemphigus vegetans with disorders presence of eosinophils contains many CLCs [129]. CLCs have also been found in the langerhans cells in histiocytosis [80].

Although CLCs are present in various diseased organs and body fluids, there are still many open questions regarding CLC formation. Perhaps CLC formation is artefactual, simply resulting from the enrichment and disruption of eosinophils. As mentioned above (See Crystallization studies of Gal-10), Gal-10 can be crystallized in a very short time under many conditions. The actual reasons for various diseases may be related to disorders of eosinophils.

## 12. Conclusions and Prospective

It has been more than 160 years since CLCs were reported. CLCs were initially identified in vivo as protein crystals that can spontaneously form in the human body. Since their discovery, CLCs have been extensively investigated. Eosinophils can over-express massive amounts of Gal-10, and Gal-10 can occasionally form CLCs in eosinophils, and by forming CLCs, the ligand binding capacity of Gal-10 may be altered. In this way, eosinophils may protect themselves from excessive stimulation. Upon an infection, eosinophils can respond to pathogens and parasites. The stimulation signals from the human body or foreign substances might trigger massive eosinophil release of Gal-10. Moreover, toxic substances released from pathogens and parasites may also induce eosinophil apoptosis or necrosis. When large amounts of Gal-10 interact, they can form CLCs in a very short time. Therefore, formation of CLCs is related to disorder of eosinophils, indicating that CLCs may be used as a potential marker for these diseases.

CLCs can rapidly form in vivo and in vitro. This greatly facilitates crystallographic studies of Gal-10. Crystallographic and biochemical studies show that Gal-10 forms dimeric structures that are significantly different from other proto-type galectins. Gal-10 forms its dimer via the S-faces of two CRDs, whereas other prototype galectins dimerize to expose their sugar binding S-faces. This unique Gal-10 S-face to S-face dimer structure may influence its ligand binding specificity, although this is little understood. 

With the development of TEM, immunological techniques and various staining methods, distributions of Gal-10 in lymphocytes, tissues and body fluids have been clarified. Gal-10 can exist in the nucleus, cytoplasm, granules, and the extracellular matrix. However, the in vivo molecular mechanisms of action for Gal-10 remain poorly understood. Only recently, has the suppressive effects of Gal-10 on T cells been revealed. Gal-10 can bind to unknown glycans on the T-cell membrane to downregulate T-cell function. The ligands for Gal-10 in the nucleus, cytoplasm, and granules are unknown. The molecular weight of Gal-10 is 16.5 kDa, which is lower than the cut off for nuclear hole transportation restrictions. This implies that cytoplasmic Gal-10 may passively diffuse into the nucleus. Interestingly, Gal-10 seems to be concentrated in the nucleus. The accumulation of Gal-10 in the nucleus suggests that this lectin may control some unknown gene expression and function. Ligands for Gal-10 in the cytoplasm are also unknown. In eosinophils, Gal-10 may be transported into primary granules. However, the transportation pathway(s) is unclear. Overall, the cellular ligands, including carbohydrates and proteins, for Gal-10 are unknown, and identification of these ligands may help our understanding of the disorder of eosinophils and even help ameliorate various disease states.
